# Factors associated with multidrug-resistant tuberculosis (MDR-TB) in Bhutan: A nationwide case-control study

**DOI:** 10.1371/journal.pone.0236250

**Published:** 2020-07-27

**Authors:** Chador Tenzin, Natkamol Chansatitporn, Tashi Dendup, Tandin Dorji, Karma Lhazeen, Dorji Tshering, Thinley Pelzang

**Affiliations:** 1 Bumthang General Hospital, Ministry of Health, Royal Government of Bhutan, Bumthang, Bhutan; 2 Department of Biostatistics, Faculty of Public Health, Mahidol University, Bangkok, Thailand; 3 School of Health and Society, Faculty of Social Sciences, University of Wollongong, Wollongong, NSW, Australia; 4 Department of Medical Services, Ministry of Health, Royal Government of Bhutan, Kawangjangsa, Thimphu, Bhutan; 5 Department of Public Health, Ministry of Health, Royal Government of Bhutan, Kawangjangsa, Thimphu, Bhutan; 6 Central Regional Referral Hospital, Ministry of Health, Royal Government of Bhutan, Gelephu, Bhutan; 7 Phuentsholing General Hospital, Ministry of Health, Royal Government of Bhutan, Phuentsholing, Bhutan; Médecins Sans Frontières (MSF), SOUTH AFRICA

## Abstract

**Background:**

Multidrug-resistant tuberculosis (MDR-TB) is a growing public health concern globally. In Bhutan, the rates of MDR-TB are high. Data on the risk factors of MDR-TB that can help inform policies are limited in Bhutan. This study aimed to determine the risk factors associated with MDR-TB.

**Methods:**

A nationwide unmatched case-control study was conducted that included 79 MDR-TB cases and 118 controls. Data was collected by trained health workers through interviews using a structured questionnaire. Logistic regression analysis was performed to identify the risk factors associated with MDR-TB.

**Results:**

The mean age of the participants was 32.4 and 33.7 years among the cases and the controls, respectively. In the multivariate analysis, the odds of having MDR-TB was higher among those who slept for less than 9 hours a day (AOR: 2.77, 95%CI: 1.11–6.92), frequently travelled in public transport (AOR: 2.96, 95% CI: 1.36–6.48), and had previous TB treatment (AOR: 5.90, 95%CI: 2.55–13.64). A greater number of rooms was also marginally associated with odds of having MDR-TB.

**Conclusions:**

The findings suggest previous TB treatment, inadequate sleep duration, and travelling by public transport to be the risk factors associated with having MDR-TB in Bhutan. Intensification of early case detection, strengthening directly observed treatment strategy, improving treatment adherence, and increasing awareness can help control the rising MDR-TB epidemic.

## Introduction

Globally, in 2017, 10 million people had tuberculosis (TB) and around 1.3 million died from TB [1]. A majority (around 46%) of the cases occurred in the South-East Asia region [2]. Approximately 3.6% of the new cases and 17% of the previously treated cases developed multidrug-resistant TB (MDR-TB) in 2017 [1]. The prevalence of MDR-TB among new and previously treated cases was 1.6% and 29% respectively, in the South-East Asia region [2]. MDR-TB is difficult to treat, requiring longer treatment duration and hospitalization, and the drugs used are less effective, costly and produce severe side effects [3]. The treatment outcome is also not always favorable [3, 4]. Thus, the prevention of initial infection, treatment and management of drug-susceptible TB adequately are keys to containing the spread of MDR-TB.

Bhutan is a small mountainous landlocked country in South Asia with a population of 727,145 [5]. Health care is delivered free of charge by the state through its three-tiered health care system that comprises of 186 Primary Health Centers (PHC) at the local level, 50 hospitals and three referral hospitals. The National Tuberculosis Control Program (NTCP), established in 1986 is responsible for the prevention and control of TB in Bhutan. All hospitals are designated as TB reporting and treatment centers. Individuals with symptoms of TB are referred from PHC to these centers for diagnosis, registration, and initiation of treatment. All confirmed TB cases are routinely tested for drug resistance and those cases found resistant to both isoniazid and rifampicin are classified as MDR-TB cases [6, 7].

Bhutan adopted the Directly Observed Treatment Short Course (DOTS) strategy in 1997. Tuberculosis patients are admitted in the treatment centers for the first two weeks for initiation of DOTS and are then followed-up by the respective health centers [6, 7]. For MDR-TB, cases are admitted in the treatment centers for the initiation of DOTS until two consecutive monthly culture results are negative [7]. A DOT provider, usually a family member is identified to provide DOTS for the remaining phase of the treatment.

Bhutan has been able to make commendable progress in TB control. This is reflected in the decline of TB prevalence from 536 in 2005 [8] to 190 per 100,000 population in 2014 [9], and high treatment success rate (more than 90% since 2005) [6, 8]. However, the drug resistance survey conducted in 2014 reported alarming rates of MDR-TB, 10.1% and 37.2% among new and previously treated cases respectively, which is much higher than the global rates [10]. MDR-TB cases have also increased over recent years [1, 6]. This suggests high transmission rates and poses a massive challenge towards achieving the Sustainable Development Goals by 2030 and the END TB targets in 2035 [11]. Urgent efforts are needed to control the MDR-TB epidemic.

Data on factors determining MDR-TB risk is limited in Bhutan. A descriptive study that recruited 19 MDR-TB patients from a treatment center of Bhutan showed that a majority of the cases did not comply with the DOT approach, had previous TB treatment history, and contact with MDR-TB cases [12]. However, this study did not assess socioeconomic and environmental factors such as education, income, housing conditions, residential area, and other lifestyle factors. Furthermore, the study was limited to MDR-TB cases in just one treatment center, did not conduct regression analysis to identify the most important factors associated with MDR-TB. More studies with better approaches that also investigates a range of plausible factors are needed to inform prevention and control policies adequately. The objective of this nationwide case-control study was to examine the factors associated with MDR-TB. The findings are expected to provide evidence for targeted policy investments that can help reduce the burden of MDR-TB in Bhutan.

## Methods

### Study design and sample

An unmatched nationwide case-control study that included all 20 districts in the country was conducted to examine the risk factors associated with MDR-TB in Bhutan. All MDR-TB patients confirmed through culture and drug sensitivity testing and receiving treatment during the study period, as per the record maintained by the national program constituted the cases. Similarly, the controls were all registered smear-positive cases, both new and previously treated, but non MDR-TB, under treatment for at least 2 months. The initial plan of recruiting two controls for every case could not be realized since there were fewer controls that fulfilled the selection criteria in some of the centers, such as Zhemgang, Gasa, and Trashiyangtse.

### Inclusion and exclusion criteria

This study included confirmed and registered TB and MDR-TB patients put on treatment during the study period. Those individuals who were 18 years or older, understood the study objectives, and were willing to participate were included. Those who were physically very ill, mentally unsound, non-Bhutanese, and were unable to communicate well were not included.

### Data collection

Data collection in the field took place from the 4^th^ of April until 28^th^ of April, 2017. A structured questionnaire with close-ended questions was used to collect information through face to face interviews from both the cases and controls. Most of the questions (25/30) used in this study were extracted from previous studies [13–17] and adapted for the current study. The selection of questions and/ or variables for the present study was informed by the literature on factors found associated with MDR-TB and their relevancy to the Bhutanese context. The questionnaire was translated into the national language “Dzongkha” and then pretested among 10 cases and 20 controls from two TB centers. Cronbach’s alpha coefficient was used to determine the reliability of the instrument for measuring stigma and knowledge [18]. The overall Cronbach’s alpha coefficient was 0.72 for knowledge and 0.73 for stigma indicating good scale reliability.

The participants were approached in their respective TB centers by the data collectors and were explained about the study. Signed informed consent was then obtained from those who were willing to participate and met the inclusion and exclusion criteria. All the cases and controls registered in each treatment centers were called one day earlier from the actual date of the interview and encouraged to participate in the study. A few participants who were unable to come to the TB centers were interviewed at home by the enumerators.

Four health workers trained on the conduct of the study, including data collection and communication with patients, collected the data. To ensure consistency and completeness of the data, the data collection process was monitored closely in the field. Data collectors were divided into two groups. One group was supervised by the principal investigator and the other by a laboratory officer. Each interview was monitored by the supervisors to ensure consistency and the completeness of data was checked at the end of the interview.

### Study variables

A wide range of variables was considered in this study that was categorized into sociodemographic, lifestyle and behavior, health, environmental, knowledge and stigma on MDR-TB. The sociodemographic factors were age, gender, marital status, education, employment status, and monthly family income. Factors such as smoking, alcohol consumption, and sleep duration and travel behavior comprised the lifestyle and behavior group. The health factors included history of TB treatment, adverse drug reaction, history of contact with TB case, BCG vaccine status, and access to health services. Area of residence, whether resided in districts bordering the neighboring country (India), frequency of traversing across the border, residential surrounding, residential house type, number of rooms, and frequency of visiting crowed areas constituted the environmental factors.

Stigma and knowledge comprised the last group. Social stigma related to the disease was measured using questions such as getting ignored, avoid talking about TB, fear of TB, feel ashamed and hurt by people’s reaction, hide TB status and change in behavior of family members. The variables were categorized into a low, moderate, and high level of stigma. The knowledge on MDR-TB was assessed using questions on awareness, mode of transmission of MDR-TB, signs and symptoms, treatment adherence and duration, and prevention. The scores against each question were used to categorize into high (≥8 scores), good (6–7 scores), and low (≤5 scores) knowledge.

### Ethical approval

The ethical clearance for this study was accorded by the Ethical Review Committee for Human Research, Faculty of Public Health, Mahidol University, and the Research Ethics Board of Health, Ministry of Health, Royal Government of Bhutan.

### Data analysis

All information in the questionnaires were entered into the record file created in Epi-data program and the data was analyzed using STATA version 14 package. Frequencies and descriptive statistics were used to describe the characteristics and distribution of MDR-TB by the explanatory variables. Logistic regression analysis was conducted to examine the presence of association between the dependent variable (MDR-TB) and the explanatory variables. Those variables found significant at the 20% level (p-value≤0.20) in the bivariate analysis were further analyzed in the multiple logistic regression model. The final model was built using the backward elimination approach. Some factors deemed to be important, as informed by the literature, were also included in the final model to reassess their effect. The Pearson χ^2^ goodness-of-fit test was performed to assess how well the final model fits the data, and multi-collinearity was assessed using the variance inflation factor (VIF). A p-value of <0.05 in the multivariable analysis was considered statistically significant.

## Results

A total of 197 participants (79 cases and 118 controls) from all the 20 districts (nationwide) in the country formed the final study sample. Of 101 MDR-TB cases registered with the NTCP and currently on treatment, 8 of them were less than 18 years of age, one could not communicate well, 2 refused to participate, 3 were mentally unsound, and 8 patients could not be traced due to the short study period. The mean age was 32.4 (SD: 2.44) years in the cases and 33.7 (SD: 3.22) years in the controls. Most of the participants were married, unemployed, and below the age of 49 years (Table 1). In both the case and control groups, a majority were 18–29 years of age, female, resided in urban areas, were frequent travelers, had middle-secondary level education, and had a family income of more than Ngultrum 10,000 per month (One USD = approximately Nu.70/-).

**Table 1:** Distribution of cases (MDR-TB) and controls (non MDR-TB) by the risk factors.

Variables	Categories	Total, N	Cases, N (%)	Controls, N (%)
**a) Sociodemographic**		N = 197	N = 79	N = 118
Age	18–29	105	44 (55.7)	61 (51.7)
	30–39	44	17 (21.5)	27 (22.9)
	>39	48	18 (22.8)	30 (25.4)
Gender	Male	85	33 (41.8)	52 (44.1)
	Female	112	46 (58.2)	66 (55.9)
Marital status	Single	67	31 (39.2)	36 (30.5)
	Married	113	42 (53.2)	71 (60.2)
	Separated/divorced	12	4 (5.1)	8 (6.8)
	Widowed	5	2 (2.5)	3 (2.5)
Education level	No education	49	18 (22.8)	31 (26.3)
	Middle secondary	88	36 (45.6)	52 (44.1)
	Higher secondary	33	13 (16.5)	20 (16.9)
	Degree and above	27	12 (15.2)	15 (12.0)
Employment status	Unemployed	66	29 (36.7)	37 (31.4)
	Student	34	16 (20.3)	18 (15.3)
	Government employee	33	13 (16.5)	20 (16.9)
	Private/others	64	21 (26.6)	43 (36.4)
Monthly income in Nu. ^&^	No income	20	10 (12.7)	10 (8.5)
	<5000	22	7 (8.9)	15 (12.7)
	5000–10,000	22	8 (10.1)	14 (11.9)
	10,000–15,000	61	27 (34.2)	34 (28.8)
	>15,000	72	27 (34.2)	45 (38.1)
**b) Lifestyle and behavior**				
Smoking (prior TB)	Yes	53	20 (25.3)	33 (28.0)
	No	144	59 (74.7)	85 (72.0)
Number of cigarettes (per day)	≤10	47	18 (90.0)	29 (87.9)
	11–20	4	0 (0.0)	4 (12.1)
	>20	2	2 (10.0)	0 (0.0)
Consume alcohol	Yes	105	38 (48.1)	67 (56.8)
	No	92	41 (51.9)	51 (43.2)
Sleep duration (hours/day)	≤8	141	63 (79.7)	78 (66.1)
	>8	56	16 (20.3)	40 (33.9)
Frequent travel in-country	Yes	137	54 (68.4)	83 (70.3)
	No	60	25 (31.6)	35 (29.7)
In-country travel mode	Private car/other	58	17 (27.0)	41 (44.1)
	Public transport	98	46 (73.0)	52 (55.9)
**c) Health**				
Previous TB treatment	Yes	50	33 (41.8)	17 (14.4)
	No	147	46 (58.2)	101 (85.6)
Number of TB treatment	1 time	33	18 (54.5)	15 (88.2)
	≥2 time	17	15 (45.5)	2 (11.8)
Adverse effect experience	Yes	15	11 (33.3)	4 (23.5)
	No	35	22 (66.7)	13 (76.5)
Treatment interruption	Yes	3	2 (6.1)	1 (5.9)
	No	47	31 (93.9)	16 (94.1)
History of TB contact	Yes	96	38 (48.1)	58 (49.2)
	No	101	41 (51.9)	60 (50.8)
BCG scar	Yes	160	62 (78.5)	98 (83.1)
	No	37	17 (21.5)	20 (16.9)
Time to nearest health center	<1 hour	161	64 (81)	97 (82.2)
	1–2 hours	23	10 (12.7)	13 (11)
	>2 hours	13	5 (6.3)	8 (6.8)
**d) Environmental**				
Place of residence	Rural	69	26 (32.9)	43 (36.4)
	Urban	128	53 (67.1)	75 (63.6)
Live near India	Yes	110	43 (54.4)	67 (56.8)
	No	87	36 (45.6)	51 (43.2)
Cross border	Frequently	42	18 (41.9)	24 (35.8)
	Occasionally/never	68	25 (58.1)	43 (64.2)
Type of residence	Multi-storied	75	37 (46.8)	38 (32.2)
	TBH & others	122	42 (53.2)	80 (67.8)
Home surrounded by buildings	Yes	123	56 (70.9)	67 (56.8)
	No	74	23 (29.1)	51 (43.2)
Number of rooms in the house	1	25	9 (11.4)	16 (13.6)
	2	36	14 (17.7)	22 (18.6)
	3	52	21 (26.6)	31 (26.3)
	>3	84	35 (44.3)	49 (41.5)
Frequent crowded areas	Daily	96	36 (45.6)	60 (50.8)
	Once a month	48	23 (29.1)	25 (21.2)
	Rarely/never	53	20 (25.3)	33 (28)
**e) Stigma and knowledge**				
Stigma level	Low (<30)	173	66 (83.5)	107 (90.7)
	Moderate (30–39)	23	13 (16.5)	10(8.5)
	High (≥ 40–50)	1	0 (0)	1 (0.8)
Knowledge level	Low (≤5)	67	21 (26.6)	46 (39)
	Good (6–7)	44	16 (20.3)	28 (23.7)
	High (≥8)	86	42 (53.2)	44 (37.3)

^&^One USD = ~Nu.70/-; TBH: traditional Bhutanese houses; ^#^towns in Bhutan bordering the neighboring country India.

The bivariate analysis revealed sleep duration of less than 8 hours per day, in-county travel by public transport, history of TB treatment, number of TB treatment, living in multistoried houses, and having residence surrounded by buildings to be associated with MDR-TB (Table 2). The multivariate logistic regression analysis showed sleep duration, history of TB treatment, travel by public transport, and number of rooms to be the predictors of MDR-TB (Table 2). TB patients who slept less than 8 hours a day were more likely to have MDR-TB than those who slept more than or equal to 8 hours (AOR: 2.77, 95%CI: 1.11–6.92). Those who traveled in public transport within the country (AOR: 2.96, 95%CI: 1.36–6.48) were also at a higher odd of developing MDR-TB than those who travelled in private cars. Relative to those not previously treated for TB, those who were previously treated for TB had higher odds of having MDR-TB (AOR: 5.90, 95%CI: 2.55–13.64). Having more than 2 rooms in the house also emerged to be marginally associated with having MDR-TB (p = 0.049). The goodness-of-fit test did not show any evidence for the lack of fit of the final model (p = 0.269). The VIF values of all the variables were less than 10 in the final model suggesting that multi-collinearity was not a concern in the analysis.

**Table 2:** Bivariate and multivariable analysis of risk factors associated with MDR-TB.

Variables	COR (95% CI)	p-value	AOR (95% CI)	p-value
**a) Sociodemographic**				
*Age* (ref: 40–77)				
18–29	1.20 (0.59–2.42)	0.61		
30–39	1.05 (0.45–2.44)	0.91		
*Gender* (ref: Male)				
Female	1.10 (0.62–1.95)	0.75		
*Marital status* (ref: Married)				
Single/previously married*	1.33 (0.75–2.37)	0.33		
*Education level* (ref: ≥Degree)				
No education	0.73 (0.28–1.89)	0.51		
Middle secondary	0.87 (0.36–2.07)	0.75		
Higher secondary	0.81 (0.29–2.28)	0.69		
*Employment status* (ref: Employed)				
Unemployed	1.45 (0.77–2.76)	0.25		
Student	1.65 (0.75–3.64)	0.22		
*Monthly income in Nu*. ^&^ (ref: ≥5000)				
<5000	1.02 (0.51–2.04)	0.96		
**b) Lifestyle and behavior**				
*Smoking* (ref: No)				
<5 cigarettes per day	1.26 (0.57–2.78)	0.57		
≥5 cigarettes per day	0.51 (0.19–1.37)	0.18		
*Consume alcohol* (ref: No)				
Yes	0.70 (0.40–1.25)	0.23		
*Sleep duration* (ref: >8 hours/ day)				
≤8	**2.02 (1.04–3.94)**	**0.04**	**2.77 (1.11–6.92)**	**0.03**
*Frequent travel in-country* (ref: No)				
Yes	0.91 (0.49–1.69)	0.76		
*In-country travel mode* (ref: Private car)				
Public transport	**2.13 (1.07–4.26)**	**0.03**	**2.96 (1.36–6.48)**	**<0.01**
**c) Health**				
*Previous TB treatment* (ref: No)				
Yes	**4.26 (2.16–8.42)**	**<0.01**	**5.90 (2.55–13.64)**	**<0.01**
*Number of TB treatment* (Ref: Nil)				
1 time	**2.63 (1.22–5.68)**	**0.01**		
≥2 times	**16.47 (3.62–74.99)**	**<0.01**		
*Adverse effect experience* (ref: No)				
Yes	1.63 (0.43–6.17)	0.47		
*Treatment interruption* (ref: No)				
Yes	1.03 (0.09–12.27)	0.98		
*History of TB contact* (ref: No)				
Yes	0.96 (0.54–1.70)	0.89		
*BCG scar* (ref: Yes)				
No	1.34 (0.65–2.76)	0.42		
*Time to nearest center* (ref:<1 hour)				
1–2 hours	1.16 (0.48–2.82)	0.73		
>2 hours	0.94 (0.29–3.03)	0.92		
**d) Environmental**				
*Place of residence* (ref: Rural)				
Urban	1.17 (0.64–2.13)	0.61		
*Live near India* (ref: No)				
Yes	0.91 (0.51–1.61)	0.75		
*Cross border* (ref: Occasionally/never)				
Frequently	1.29 (0.59–2.83)	0.52		
*Type of residence* (ref: TBH & others)				
Multi-storied	**1.85 (1.03–3.34)**	**0.03**		
*Residence surrounded by buildings* (ref: No)				
Yes	**1.85 (1.01–3.40)**	**0.04**		
*Number of rooms in the house* (ref: ≤2)				
>2	1.22 (0.51–2.92)	0.66	**2.42 (1.01–5.84)**	**0.05**
*Frequent crowded areas* (ref: Rarely/never)				
Daily	0.99 (0.49–1.98)	0.98		
Once a month	1.52 (0.69–3.36)	0.30		
**e) Knowledge and stigma**				
*Stigma level* (ref: Low)				
Moderate-high	1.92 (0.81–4.53)	0.13		
*Knowledge level* (ref: Moderate-high)				
Low	0.57 (0.30–1.05)	0.07		

COR: crude odds ratio; CI: confidence interval; AOR: adjusted odds ratio

*include separated, widowed and divorced

^&^One USD = ~Nu.70/-; TBH: traditional Bhutanese houses; ^#^towns in Bhutan bordering the neighboring country India

## Discussion

The results of this study showed that sleep duration, travel by public transport, previous TB treatment, number of TB treatment, residential house type, and having residence surrounded by buildings were associated with MDR-TB in the bivariate analysis. In the multivariate analysis, sleep duration of less than 8 hours per day, travelling in public transport, previous TB treatment, and a greater number of rooms were associated with higher odds of developing MDR-TB. To our knowledge, this is the first nationwide case-control study that examined the risk factors associated with MDR-TB in Bhutan. The findings may help inform planning and policy interventions for effective control and prevention of MDR-TB in Bhutan.

Consistent with the evidence in the literature [13, 15, 19–21], those who were previously treated for TB had higher odds of developing MDR-TB. The number of times treated for TB also emerged to be positively associated with MDR-TB in the bivariate analysis. Incomplete treatment due to poor adherence to TB treatment and erratic drug supply, and inappropriate treatment regimens in previous treatment episodes can result in the emergence of MDR strains to any TB drugs [16, 19, 21, 22]. Inadequate financing and technical capacity, and poor TB program supervision also contributes to poor case management, leading to increased morbidity [3]. In Bhutan, suboptimal practice and adherence to DOTS, inadequate supervision and human resources, and poor infection control were highlighted as some of the programmatic challenges [9]. Rigorous patient follow-up and monitoring, improving patient counselling and communication, alternative supervision and choice of DOTS provider, and family member education may help promote compliance [23]. Future studies that investigate factors influencing retreatment and treatment failure, and deeper exploration of DOTS provision may help inform MDR-TB prevention efforts.

The results also indicated that those who frequently travel in public transport such as buses and taxis were more likely to develop MDR-TB than those travelling via private vehicles. The results suggest the free movement of infectious sources in such public spaces and late case detection leading to increased transmission. Our finding is in agreement with a study that reported a positive association between public transport workers and MDR-TB risk [14] and another one that showed higher TB risk among public transport users [24]. Likewise, a study also found higher odds of MDR-TB among frequent travelers [17]. Crowding, proximity to the index case and longer duration of exposure can amplify the risk of transmission in such settings. Furthermore, generally people from the low-income groups who cannot afford to own cars may be the ones using public transport, and these individuals are also more likely to have TB/MDR-TB [19, 22, 25]. Those in the lower socioeconomic group also have low education and poor housing conditions and may be unemployed that exacerbates their MDR-TB risk [15, 25]. Developing partnership with public transport providers aimed to raise awareness on infection control and motivate referral of symptomatic individuals may reinforce early case detection efforts.

Our analysis also revealed that those who slept for less than 8 hours a day were more likely to have MDR-TB. In contrast, the association was not robust in a few studies [25, 26]. A prolonged period of inadequate sleep can lead to chronic stress, which in turn affects the immunity through upregulation of stress hormones and downregulation of immunity. The dysregulation of body physiological system perpetuated by exposure to stressors and unhealthy behaviors including poor sleep also called “allostatic load,” can also expedite the development of chronic diseases [27, 28], that in turn can potentially increase susceptibility to TB through immune system impairment. This may partly help explain the association between sleep and MDR-TB. Studies have also shown that those with MDR-TB were more likely to endure life pressure or stress and have poor mental health [26, 29]. It is possible that patients may experience sleep difficult due to the physical and psychological trauma and social stressors associated with MDR-TB [29]. The relationship between sleep and MDR-TB warrants further investigation with better study design and data.

The results also showed having a greater number of rooms to be marginally associated with MDR-TB. The greater number of rooms among those with larger family size in this study sample can be a possible explanation for this finding. The proportion of having more than three rooms were 47.1%,47.6% and 40.3% among those with 3, 3–5 and more than 5 family members respectively in this study sample. Greater family size and household crowding has been shown to be associated with TB risk [30, 31]. Larger family size may have increased contacts among the household members, including with index cases, resulting in a higher propensity of exposure and transmission. Although, these findings, including for sleep duration seems plausible, they may also be statistical artefacts representing other correlated factors.

The absence of strong statistical associations for other factors, however, does not mean that they are not important. Variations in the epidemiology of MDR-TB and these factors may have led to the non-robust association. Besides, the small sample might have also reduced the power of the study to detect significant associations. Furthermore, the influence of factors such as social support and connectedness, substance abuse [32], mental health [22], HIV status [20, 32], clinical and laboratory manifestation of TB (such as quantity of bacilli in sputum smear/ smear positivity during course of treatment, lung cavity features, pleural effusion) [16, 21] were not assessed in our study. Future studies need to consider these factors to gain a deeper understanding of the factors influencing MDR-TB in Bhutan.

### Strengths and limitations

The strengths of the study were the inclusion of a majority (around 78%) of the total MDR-TB cases on treatment during the study period. Thus, the findings are widely applicable and can inform policy interventions that may help curb the rising burden of MDR-TB in Bhutan. We also assessed a wide range of plausible factors, and the results can be a good addition to the body of evidence on the risk factors of MDR-TB. This study also had some limitations, including those inherent to case-control designs. First, the findings are prone to recall bias that could have potentially reduced the strength of association since most of the information obtained was of exposure that occurred prior to illness. Second, the inability to blind the interviewers who were also health workers could have biased the results to some extent. Although the effect may be trivial, those patients who could not be interviewed could be different from the participants. Since this study used an unmatched design, the confounding effect of some factors cannot be ruled out. Finally, we were not able to identify adequate controls that might have potentially reduced the power to detect the effect of some risk factors.

## Conclusions

This nationwide case-control study revealed previous TB treatment, use of public transport for travelling, inadequate sleep, and number of rooms to be associated with a higher risk of having MDR-TB. The findings suggest that identifying the source of infection through early case detection and effective management of drug-susceptible cases, implementing strategies to enhance adherence and improve infection control are essential in halting and reducing the rising MDR-TB burden.

## Supporting information

S1 File Questionnaire. (PDF)
