# Supplementary material for: Factors associated with multidrug-resistant tuberculosis (MDR-TB) in Bhutan: A nationwide case-control study
Source: PLoS One. 2020 Jul 27;15(7):e0236250. doi: 10.1371/journal.pone.0236250 (PMC7384628; doi:10.1371/journal.pone.0236250)
Supplement: S1 File — (PDF) [file pone.0236250.s001.pdf]

Appendix A - Questionnaire

Research title: Determinants of multi-drug resistant Tuberculosis in Bhutan

Please mark (✓) in the box provided or fill in the blanks (...……..…) with the most appropriate answer known to the best of their knowledge.

Name of interviewer: ………………………………

Date of interview: ………………………………….

1.0 Patient's general characteristics

1.1	How old are you?<br>………………years.	
1.2	What is your gender?<br>☐ Male	 ☐ Female
1.3	What is your current marital status?<br>☐ Single<br>☐ Widowed<br>☐ Divorced	 ☐ Married<br>☐ Separated
1.4	What is the highest level of education attained?<br>☐ Illiterate<br>☐ Lower secondary school<br>☐ Higher secondary school<br>☐ Others…………………………………..	 ☐ Primary school<br>☐ Middle secondary school<br>☐ Bachelor's degree and higher
1.5	What is your current occupation?<br>☐ Unemployed<br>☐ Uniform personnel<br>☐ Private or self-employed	 ☐ Student<br>☐ Government employee<br>☐ Others………………………………….
1.6	What is your monthly family income?<br>☐ None<br>☐ Nu. 5000-10000<br>☐ More than Nu. 15000	 ☐ Less than Nu. 5000<br>☐ Nu. 10000-15000

2.0 Lifestyle and behavior

2.1	Did you smoke before getting TB?<br>☐ No, if no go to Q. 2.3	 ☐ Yes
2.2	If yes, how many cigarettes did you smoke per day?<br>☐ Less than 5/day<br>☐ 5-10/day	 ☐ 11-20/day<br>☐ More than 20/day
2.3	Did you drink alcohol?<br>☐ No	 ☐ Yes
2.4	Do you get enough sleep?<br>☐ Yes	 ☐ No

2.5	How many hours do you sleep in a day?<br>☐ Less than 6 hours ☐ 6-8 hours<br>☐ 9-10 hours ☐ More than 10 hours
2.6	Do you frequently travel within the country?<br>☐ Yes ☐ No
2.7	What mode of transport do you use the most?<br>☐ Private vehicle ☐ Public transport (Bus and Taxi)<br>☐ Others…………………………………

3.0 History of TB treatment

3.1	Have you ever received TB treatment before?<br>☐ No, if no go to Q. 3.5 ☐ Yes
3.2	If yes, how many times were you treated for TB?<br>☐ Once ☐ Two times<br>☐ Three times ☐ More than three times
3.3	Did you have any side effects with the medicine during the previous treatment?<br>☐ No, if no go to Q. 3.5 ☐ Yes
3.4	Did you have to stop/interrupt the treatment due to side effects?<br>☐ No ☐ Yes
3.5	Did you live with anyone who was sick of TB in the past?<br>☐ Yes ☐ No
3.6	Do you have a BCG scar? (check on the right arm)<br>☐ Yes ☐ No
3.7	How many hours does it take to reach the nearest health center?<br>☐ Less than 1 hour ☐ 1-2 hours<br>☐ More than 2 hours

4.0 Environment

4.1	Where did you live the most in the past three years?<br>☐ Rural ☐ Urban<br>☐ Semi-Urban
4.2	Did you stay/live near the border to India for more than one month in the last 3 years?<br>☐ No, if no go to Q. 4.4 ☐ Yes
4.3	How often do you go across the border?<br>☐ Daily ☐ Occasionally<br>☐ 2-3 times a week ☐ Once a week<br>☐ Never crossed the border
4.4	What type of building do you live in?<br>☐ Multistoried building ☐ Bungalow type<br>☐ Traditional Bhutanese house ☐ Others (specify)………………………..
4.5	The building you live in is surrounded by? (choose more than one answer if applicable)<br>☐ Buildings only ☐ Agricultural field<br>☐ Plants and trees ☐ None of the above<br>☐ Others (specify)……………………

4.6	How many rooms do you have in your house?<br>☐ One room ☐ Two rooms<br>☐ Three rooms ☐ More than three rooms
4.7	How many family members are there in your family?<br>☐ 1 ☐ 2<br>☐ 3 ☐ 4-5<br>☐ More than 5
4.8	How often do you go to crowded places?<br>☐ Everyday ☐ 2-3 days in a week<br>☐ Once a month ☐ Rarely

5.0 Social stigma

No	Statement/s	Strongly Agree	Agree	Uncertain	Disagree	Strongly disagree
1	I feel ashamed because I have TB					
2	My family hide the fact that I have TB from the community					
3	I hide TB status from the community					
4	I avoid talking about TB in the presence of other family members or neighbors					
5	I am afraid that someone will see me at the health care clinic where I am being treated					
6	I substitute another word for TB in the conversations with my friends					
7	I have noticed changes in my family member's behavior towards me since the TB diagnosis					
8	Close friends of mine ignore our friendship when they know about my TB status					
9	I am hurt by some people's reaction when they know about my TB status					
10	People fear for those having TB					

6.0 Knowledge on MDR-TB

6.1	Have you heard of MDR-TB?<br>☐ No ☐ Yes
6.2	What is MDR-TB?<br>☐ It is same as normal TB ☐ Disease caused by virus<br>☐ TB which is easier to treat than normal TB ☐ TB which can't be cured with standard treatment

6.3	Is MDR-TB a transmissible disease?<br>☐ No ☐ Yes<br>☐ Don't know
6.4	How can a person get MDR-TB?<br>☐ Through handshakes ☐ Through eating from the same plate<br>☐ Through the air when a person with TB coughs or sneezes ☐ Through sharing dishes<br>☐ Do not know
6.5	How does normal TB become MDR-TB?<br>Because of:<br>☐ Poor nutrition ☐ HIV<br>☐ Not taking TB treatment properly ☐ Witchcraft<br>☐ Don't know
6.6	Which is the commonest symptom of MDR-TB?<br>☐ Headache ☐ Vomiting<br>☐ Cough ☐ Diarrhea
6.7	How long is the duration of MDR-TB treatment?<br>☐ Three months ☐ Six months<br>☐ One year ☐ More than one year
6.8	What is the duration of cough regarded as TB symptom?<br>☐ Less than one week ☐ One week<br>☐ More than two weeks ☐ One month<br>☐ One year
6.9	How can a person prevent getting TB?<br>☐ Avoid shaking hands ☐ Covering mouth and nose when coughing or sneezing<br>☐ Avoid sharing dishes ☐ Washing hands after touching items in public places<br>☐ Closing windows at home ☐ Through good nutrition<br>☐ By praying
6.10	How can someone with TB be cured?<br>☐ Praying ☐ Herbal remedies<br>☐ Home rest without medicine ☐ Specific drugs given by health center<br>☐ Don't know
6.11	If the symptoms of TB are resolved, can we stop taking medicine even if the prescribed treatment duration has not been reached/is not complete?<br>☐ No ☐ Yes

………………………………………………*End*……………………………………………….....
